# Of pigs and men: the best-laid plans for prevention and control of swine fevers

**DOI:** 10.1093/af/vfaa052

**Published:** 2021-02-05

**Authors:** Jishu Shi, Lihua Wang, David Scott McVey

**Affiliations:** 1 Department of Anatomy and Physiology, College of Veterinary Medicine, Kansas State University, Manhattan, KS; 2 School of Veterinary Medicine and Biomedical Sciences, University of Nebraska Lincoln, VBS, Lincoln, NE

**Keywords:** African swine fever, classical swine fever, eradication, infectious disease, vaccine

**Implications**

- Know your enemy (the disease and pathogen) through supporting innovative research. Government and the industry should invest strongly and continuously in research related to African swine fever. Important research areas include African swine fever virus (ASFV) biology, ASFV-host interaction, point-of-contact diagnostics, safe and efficacious vaccines, swine farm biosafety and biosecurity risk management systems, and high containment facilities that are suitable for African swine fever research.
- Science and technology alone are not enough without purpose and direction. All stakeholders of the swine industry should develop and enact science-based policies on foreign animal disease outbreak emergency management.
- To eradicate swine fevers, leaders of the swine industry and governments should work together. Governments should ensure their goals and policies are fully supported by swine farm owners, farm employees, pork processing plants, animal health companies, veterinarians, regulatory agencies, social media, and the public.
- The transboundary nature of emerging and re-emerging high consequence animal infectious disease threats requires global cooperation. This international cooperation should be not only in outbreak management, but also in research for a broader biomedical, social, and ecological understanding of disease systems.

## Introduction

John Steinbeck drew the title of his novel “Of Mice and Men” from a line in a Robert Burns poem “To a mouse”: “The best-laid plans of mice and men/Go often awry.” Unlike John Steinbeck who used the title to mirror the characters who were struggling during the Great Depression to the mouse whose nest was accidentally destroyed by the poet (Burns 1785), we chose this line to emphasize that the best-laid plan can go wrong in infectious disease control and prevention. Here, we will discuss the contributing factors behind the global successes and failures in the prevention and control of swine fevers—classical swine fever (CSF) and African swine fever (ASF).

## Swine Fevers (Classical Swine Fever and African Swine Fever) are not Swine Flu

Swine fevers and swine flu are different diseases caused by completely different viruses. However, swine fevers and swine flu are often regarded as the same disease by the public. This is in part due to the 2009 H1N1 influenza pandemic where the human influenza virus contained genetic segments from the swine influenza virus (Neumann et al., 2009). Swine flu and human flu are caused by negative-strand RNA viruses (influenza A virus). In contrast, CSF and African swine fever are caused by a small positive-strand RNA virus (CSF virus, CSFV) and a large double-strand DNA virus (ASF virus, ASFV), respectively. To date, no evidence suggests that ASFV and CSFV can infect humans, even though they often cause lethal infection in pigs of all ages. Various inactivated swine flu vaccines with different levels of efficacy are used on swine farms all over the world. On the other hand, safe and efficacious modified live virus (MLV) vaccines (such as the C-strain vaccine) have contributed to the successful control of CSF in many countries (Luo et al., 2014; Blome et al., 2017). But there is no safe and efficacious vaccine for ASF.

## Vaccines and Diagnostics: Technological Tools for Infectious Disease Control and Prevention

Vaccines are the most cost-effective tools for animal infectious disease control and prevention in disease-endemic regions. Based on the nature and/or production method of antigens, vaccines can be classified into five different categories: 1) tissue-derived vaccines (inactivated or live) with little or no antigen purification; 2) inactivated vaccines in which pathogens are inactivated by the chemical methods after they are processed from cell culture or fermentation systems; 3) MLV vaccines with naturally or genetically modified attenuated live microbes; 4) subunit vaccines in which the antigens are purified from native pathogen cultures or recombinant expression systems; and 5) nucleotide (DNA and RNA) vaccines in which partial genetic segments from the pathogens are used to directly induce antigen expression in the immunized animal or incorporated into microbial vectors for antigen expression and delivery.

The selection of a certain type of vaccine for field use in animal disease control and prevention should be based on its safety and efficacy profile and cost-effective analysis, not how the vaccine is produced. The first three categories (tissue-derived, inactivated, and MLV) of vaccines have been used in the field since the late 1800s and the last two categories of vaccines (subunit and nucleotide) were developed with new technologies in the last few decades (McVey and Shi, 2010). For many infectious diseases, one or more of the five types of vaccines have been developed with robust and efficient manufacturing processes. Therefore, safe and efficacious vaccines are affordable and available for use in various animal populations.

In addition to vaccines, diagnostics are also essential tools for animal disease control and prevention. For antigen/pathogen detection, antigen capture antibody enzyme-linked immunosorbent assay (ELISA), real-time quantitative polymerase chain reaction (PCR), lateral flow assay (LFA), and a fluorescent antibody test (FAT) are routinely used in a laboratory setting. Various forms (indirect, Sandwich, and competitive) of ELISA have been developed to detect antigen/pathogen-specific antibodies in animals after vaccination or infection. Virus serum neutralization assays (and surrogate assays like hemagglutination inhibition) are still very useful for characterizing antibody responses.

Diagnostics that can differentiate infected from vaccinated animals (DIVA) are crucial tools for animal disease control and eradication. DIVA assays are extremely useful for the control of a newly emerging infectious disease or a foreign animal disease as they can enable the “vaccinate-to-live” strategy by which vaccinated animals can be raised and processed for food production and consumption and/or international trade. Genetic DIVA assays are designed to identify the genetic difference between a vaccine antigen and a virulent field pathogen. Serological DIVA assays target the difference in host immune response to the vaccine strain (after vaccination) and virulent field strain (after infection).

## Classical Swine Fever/Hog Cholera

### What is classical swine fever?

Pigs with CSF, also known as hog cholera, have clinical signs such as high fever, loss of appetite, lethargy, and high mortality rate. CSF/hog cholera was first reported in the Ohio river valley in the 1830s, and it still causes significant economic losses to the swine industry in Asia and presents a significant agricultural security threat to CSF-free countries such as the United States. CSF is probably one of the earliest swine viral diseases identified by animal disease researchers in the early 20th century. It was the United States Department of Agriculture (USDA) scientists Emil Alexander de Schweinitz and Marion Dorset who first demonstrated in 1903 that the highly contagious hog cholera was caused by a virus (not a bacterium) and hogs that survived from the infection were immune from future infection (Lofflin, 2009).

### CSF control and its impact on animal health regulation in the United States

Hog cholera/CSF caused devastating losses to American swine producers since the late 1800s. According to USDA’s historical data, “Outbreaks in 1886, 1887, and 1896 each killed more than 13% of the Nation’s hogs; more than 10% died during the 1913 outbreak. The disease was still costing producers $50 million a year in the early 1960’s” (USDA 2019). Around the beginning of the 20th century, smoke rising aloft from the burning of dead pigs on farms across the prairies of the Midwest was the heart-breaking evidence of CSF destruction. It is not an overstatement that CSF was the most destructive disease of swine in the United States for more than a century (1830 to 1970).

Although the eradication of CSF from the United States in 1978 was a great success story, one must remember that many important pieces of research were carried out before the 17-yr effort (1961 to 1978), with the support from the pork industry as well as State and Federal governments. After the initial federal ban (1963) on interstate shipment of virulent CSF virus or of feeder pigs and breeding stock vaccinated with CSF vaccines, use of MLV vaccines and inactivated vaccines continued until banned in 1969 (Lofflin, 2009; USDA, 2019). Most of the control policies were developed based on the early CSF research findings of USDA scientists and veterinarians. Injection of hyperimmune anti-CSF serum plus CSF virus was used as a routine CSF control method for decades until CSF vaccines with reasonable efficacy were developed in the 1950s. Large scale field trials involving thousands of swine farms were conducted to evaluate the field efficacy of anti-CSF biologics (vaccines and antiserum products). The plans and policies for CSF eradication in the United States were developed based on the knowledge regarding how the CSFV was transmitted. Other significant contributions included clinical trials with anti-hog cholera serum products, various inactivated CSF vaccines and MLV vaccines, and the development of fast and accurate diagnostic methods for CSF.

**Figure 1.**
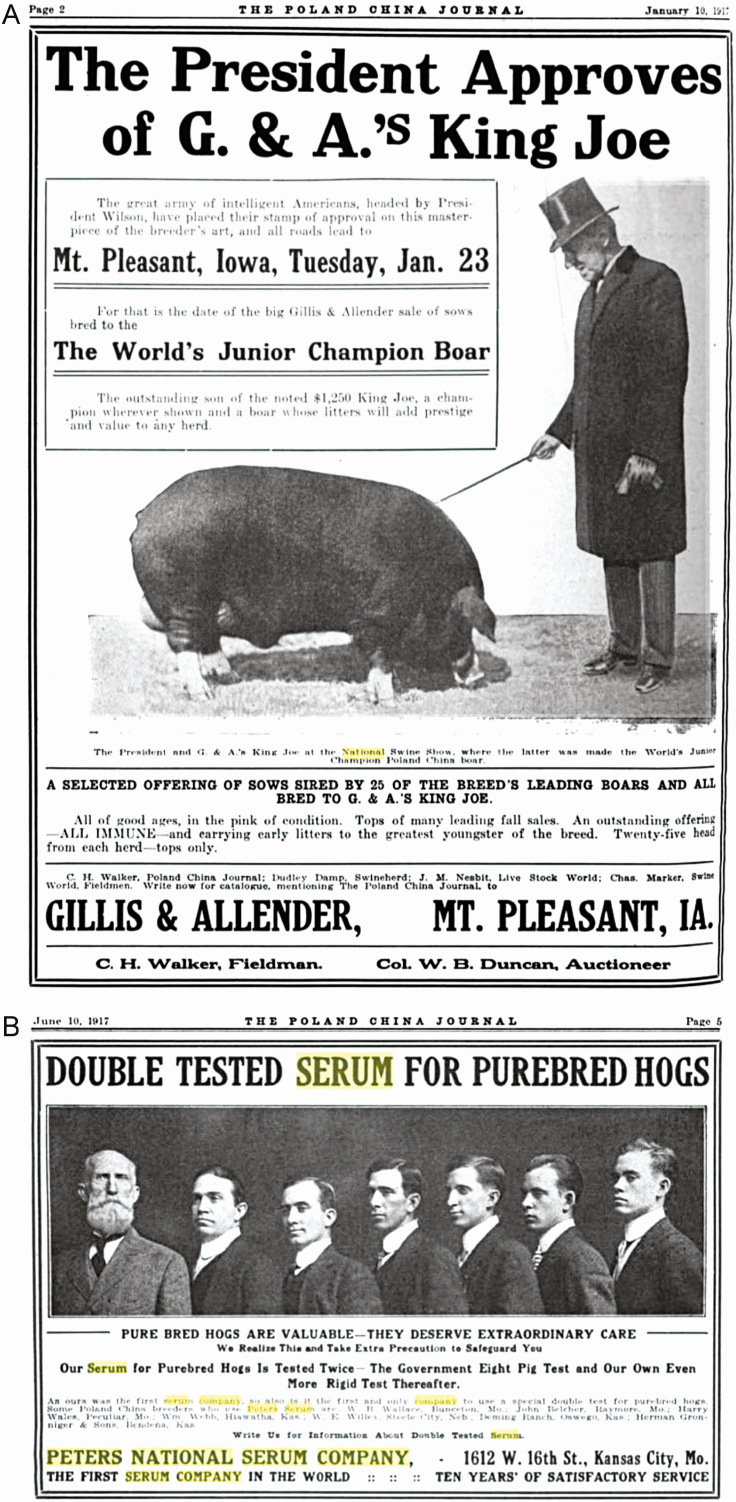
Pigs were important animals to the President and other politicians 100 yr ago. Shown are two advertisements in The Poland China Journal (January 10, 1917) that depicted the relationship between pigs and politicians in the early 20th century. (A) President Woodrow Wilson at the National Swine Show in 1916. (B) Former U.S. Congressman Mason S. Peters and his six sons formed the National Serum Company with seven serum plants around Kansas City.

Actions and governmental regulations associated with CSF control in the United States played an important role in the development of animal health policies in general. Since the discovery that pigs injected with hyperimmune serum could be protected from CSF virus challenge in 1907 (USDA, 2019), anti-hog cholera serum production and processing plants mushroomed in Kansas City and the rest of the Midwest (Lofflin, 2009). Interestingly, pigs were not only an important food source for ordinary Americans 100 yr ago, they were also very important to the politicians. Then-President Woodrow Wilson attended National Swine Show (Figure 1A), and his Secretary of the U.S. Food Administration Herbert Hoover believed that food would help the U.S. win World War I and started a national campaign for greater swine production (Nebraska, 2020). He said in 1917: “We need a ‘keep-a-pig’ movement in this country, and a properly cared for pig is no more unsanitary than a dog. Every pound of fat is as sure of service as every bullet, and every hog is of greater value to the winning of this war than a shell.”

Given the social and economic importance of pork production in the United States at the beginning of the 20th century, perhaps it was not a surprise that one of the earliest anti-hog cholera serum plants in the Kansas City area was created by Mason Peters, a lawyer and former U.S. congressman in Kansas (Kansas, 1914). He saw the potential of this biological product (Figure 1B). Mason Peters “was one of the most active in the original research work for the practical use of this remedy to combat hog cholera.” Equally amazing is that an academic institution like Kansas State Agricultural College also owned and operated an anti-hog cholera serum plant from 1908 to 1948 (Dykstra, 1952). The transgenerational significance of that serum plant location is obvious as the “Serum Plant Road” on Kansas State University campus today leads to USDA’s National Bio and Agro-defense Facility (NBAF) in which research related to CSF will continue (Montgomery, 2019).

Furthermore, the Virus-Serum-Toxin Act, which enacted federal regulation of veterinary biologics in 1913, was passed largely because of public concerns over the safety and efficacy of veterinary vaccines from Europe and hog cholera products being produced and marketed across the country (USDA, 2020). The new law required the USDA to ensure that veterinary biologics (vaccines, bacterins, antiserums, and similar products) sold in the United States are pure, safe, potent, and efficacious.

The successful eradication of CSF in the United States was the result of a determined and comprehensive approach including 1) more than 60 yr of scientific research and development on CSF virus and the disease management tools (antiserum products, vaccines, and diagnostics); 2) science-based regulatory decisions from all levels of government; and 3) the public and private partnership of all stakeholders related to the swine industry. We can summarize the best-laid plan in CSF prevention and control (the U.S. story) as:

1. Know your enemy (the disease and pathogen) through supporting innovative research.
2. Develop and implement science-based governmental policies at both state and federal levels.
3. Ensure the cooperation of all stakeholders of the pork industry including pig producers, animal health companies, veterinarians, and regulatory agencies.

### Why CSF is still endemic in Asia and how can it be eradicated in the future?

It has been clearly demonstrated that CSF can be eradicated with less ideal tools (vaccines and diagnostics) in a country with large and intensive swine production systems. Nevertheless, CSF remains one of the most devastating diseases of swine in many other large pork-producing countries such as China, Vietnam, Thailand, Japan, South Korea, and the Philippines. This phenomenon is intriguing as these countries have produced or have had access to the C-strain CSF vaccines that are affordable, available, safe, and efficacious against all known genotypes of the CSF virus.

With the development of better vaccines and faster and more accurate diagnostic assays over the last 20 yr, CSF endemic countries have more and superior technological tools for CSF control and eradication than the United States did in 1960 to 1978. Subunit vaccines based on CSFV structural protein E2 have been marketed since the 1990s and newer versions of E2 subunit vaccines have also been now manufactured and marketed by different companies in Asia (Blome, et al., 2017; Gong, et al., 2019). One of the distinct advantages of E2 subunit vaccines is their intrinsic capability of differentiating vaccinated from infected animals in which infected pigs would produce antibodies against other CSFV structural proteins such as E^rns^ (Madera et al., 2016; Wang et al., 2020).

The C-strain MLV vaccine is an attenuated live virus and can provide complete protection against wild-type CSFV with the onset of effective immunity just 5 d after vaccination (Graham et al., 2012). The only drawback of this vaccine is that it is difficult to differentiate pigs vaccinated with C-strain from pigs infected with field strains of CSFV. This shortcoming may be overcome soon because a C-strain CSFV E^rns^-specific monoclonal antibody (mAb) has been recently generated by our group (Wang et al., 2020). A cELISA is being developed to differentiate pigs vaccinated with the C-strain vaccine from pigs infected with wild-type CSFV or unvaccinated pigs, based on the observation that the latter two groups of pigs do not produce antibodies that can compete with this C-strain E^rns^-specific mAb. This is an example of a positive DIVA marker.

With the help and guidance from the Food and Agriculture Organization (FAO) and the World Animal Health Organization (OIE), many if not all CSF endemic countries in Asia have developed national policies for CSF control and eradication (China, 2012; FAO and OIE, 2014). Thus, it is not the lack of technological tools and/or government policies that have hindered the eradication of CSF in these CSF endemic countries. Because the C-strain vaccine can be cost-effectively produced and marketed or freely distributed to swine producers in China and other Asian countries, lack of resources (vaccines) does not seem to be the major constraint to control CSF, which is often the case in tackling a major disease epidemic such as the COVID-19 (McMahon et al., 2020).

There is no doubt that CSF outbreaks can be effectively controlled by routine and high coverage vaccination with the C-strain vaccine, but the success of this approach requires government support in providing sufficient and qualified field veterinarians and establishing an effective disease diagnostic and epidemic information network. More importantly, the government at all levels (central and local) should provide sufficient technical support and financial compensation to swine producers whose pigs might have to be culled due to localized CSF outbreaks. Furthermore, government, industry associations, and the media can also play an important role in raising public awareness that CSF can and should be eradicated soon. Without an effective eradication plan, CSF will continue to negatively affect general consumers due to pork price increase and overall inflation when pork production is disrupted by disease outbreaks.

Thus, the eventual eradication of CSF from CSF endemic countries may depend on whether and when all stakeholders of the pork industry can form a real partnership and work cooperatively for the same goal. To make this partnership effective, pork producers and animal health companies also must equally contribute to control and eradication efforts. These efforts will include strict compliance with government regulations on vaccination and animal movement; eliminate production, marketing, and use of CSF vaccines when a vaccination ban is placed in effect in the final stages of a CSF eradication plan.

## African Swine Fever

### What is African swine fever?

**Figure 2.**
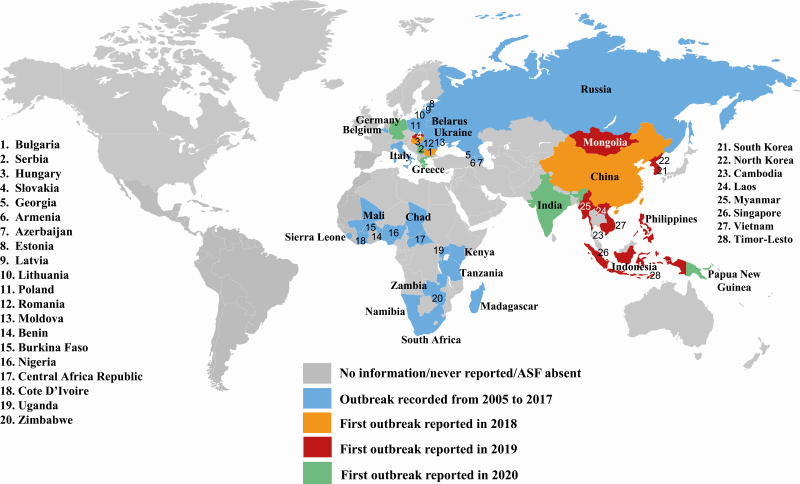
Global distribution of ASF, 2005–2020. This map is based on data from the OIE World Animal Health Information system (https://www.oie.int/wahis_2/public/wahid.php/Diseaseinformation/Diseaseoutbreakmaps?) and Global Disease Monitoring Reports (https://www. swinehealth.org/global-disease-surveillance-reports/). The names of countries with ASF are given on the map. Countries with continuing ASF outbreaks were labeled with the year when the first outbreak was reported since 2005. Data not shown: the following countries reported new ASF outbreaks in 2019: Sierra Leone, Chad, Belgium, Hungary; and in 2020: Cote d’Ivoire, Nigeria, Kenya, Zambia, Namibia, South Africa, Greece, Bulgaria, Serbia, Slovakia, Poland, Lithuania, Latvia, Estonia, Ukraine, Romania, Moldova, Russia, China, Mongolia, Myanmar, Laos, Cambodia, Vietnam, Indonesia, Timor-Leste, Philippines, South Korea, and North Korea.

Although CSF and ASF share similar clinical signs such as high fever, loss of appetite, lethargy, and high mortality rate, these two diseases are caused by two distinct and unrelated viruses. The CSFV is a small (12.3 kb) RNA virus with only four structural proteins, while the ASFV is a large DNA virus (170 to 190 kb genome) with more than 50 structural proteins (Schulz et al., 2017). Since ASF was first reported in Kenya, ASF research has been the focus for only a few laboratories in Europe after its first emergence in the 1960s. This might be partially due to the observation that ASF was eradicated in most parts of Europe in the 1990s. The re-emergence of ASF in east European counties since 2007 sparked more interest in ASF research (Borca et al., 2016), the urgency for and the intensity of ASF research are increased significantly worldwide only after the ASF outbreak was first reported in China in 2018 (Zhou et al., 2018). Since then, ASF outbreaks have occurred in many other pork producing countries in Asia including Vietnam, South Korea, Cambodia, Laos, the Philippines, and Indonesia (Figure 2). More recently, ASFV has been detected in wild boars in Belgium and Germany (USDA, 2020). Because there are significant knowledge gaps about ASFV and ASFV–host interactions, it is no surprise that safe and efficacious commercial ASF vaccines have yet to be developed.

### Was an ASF outbreak in China/Asia inevitable?

Three conditions might explain why ASF research in China was not a priority before 2018: 1) limited preparations for ASF research—there were very limited high containment (biosafety level 3) research facilities in China that were available for animal studies on foreign animal diseases such as ASF; 2) false security—CSF and foot and mouth disease (FMD), two other highly contagious and devastating swine viral diseases are largely controlled in China via mass vaccination; and 3) false optimism—because ASF has been largely eradicated in Europe in the 1990s, it was not hard to imagine that ASF could be controlled quickly by culling pigs infected with ASF virus. Consequently, research on ASF as a foreign animal disease was not carried out as a priority in China to develop the tools essential for the prevention and control of ASF.

Before the rapid spread of ASF in China that was first reported in August 2018, policymakers in China were aware of the serious threat of ASF and had implemented an ASF-specific national policy—“Technical Specification for Prevention and Treatment of African Swine Fever” in 2015 (China, 2015). Based on online public reports (https://finance.huanqiu.com/article/9CaKrnJY1uN and http://www.cpwnews.com/content-23-9199-1.html), the General Administration of Customs of China (GACC) and the Ministry of Agriculture (MOA) organized several ASF-specific emergency response drills in northern provinces and cities including Inner Mongolia, Hebei, Beijing, and Tianjin in 2016 and 2017. The risk of importing transboundary animal diseases associated with the “One Belt One Road” Initiative (BRI) was highlighted as the rationale behind these exercises.

Although no direct evidence that ASFVs were introduced to China via commercial activities of the BRI, there are two intriguing relevant observations: 1) the first ASF outbreak was likely started in mid-June (was confirmed on August 2, 2018) on a swine farm in the outskirts of Shenyang (Zhou et al., 2018), the provincial capital of Liaoning Province; and 2) on June 11, 2018, the first convoy of six trucks and two buses supplying with fruits and vegetables returned from a 25-d round trip from Dalian, China to Novosibirsk, Russia. Shenyang is 400 km from Dalian and a likely stop on the road from Novosibirsk to Dalian (https://www.sohu.com/a/238415268_267831?_f=index_pagerecom_417). However, what happened next was puzzling: the second ASF case was confirmed 12 d later in Zhengzhou (http://www.xinhuanet.com /fortune/2018-08/16/c_1123281884.htm), which is 1300 km south of Shenyang.

**Figure 3.**
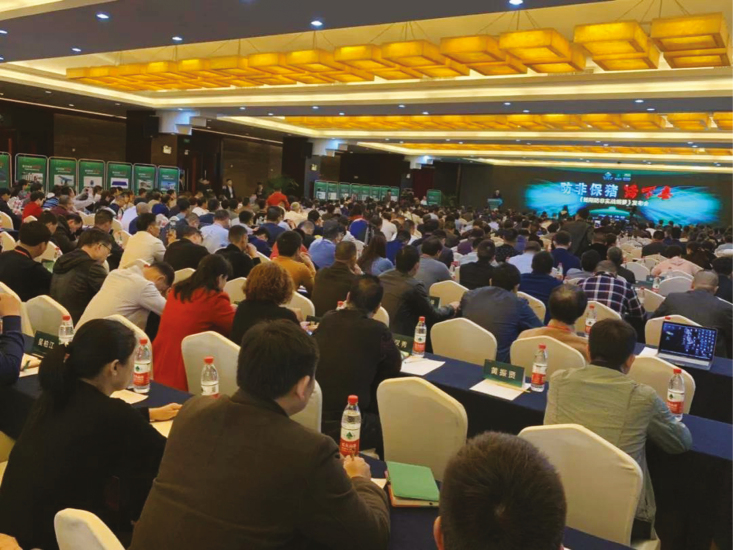
ASF meetings for swine producers were held frequently in China during the first half of 2019. Shown here was an ASF meeting at Nanning, China on March 20, 2019. While 500 people pre-registered, 800 swine farmers and animal health professionals showed up at this “Protect Pigs from ASF and Survive” meeting. The focus of this meeting was on-farm practices that can minimize the biosafety and biosecurity risks associated with swine production. Photo courtesy of Mr. Yuanfei Gao of Yangxiang Group, the organizer of this ASF meeting.

It is even more troubling and puzzling that tens of millions of pigs were lost due to ASF outbreaks all over China and some of its neighboring countries in less than 1 yr. These losses probably eclipsed the total number of pigs lost on the entire planet to ASF over the previous 90 yr. ASF meetings in China were often packed with hundreds of swine producers with the hope to find a miracle weapon to control or prevent ASF on their farms (Figure 3). Without the help of a safe and efficacious commercial ASF vaccine, swine producers in China and the rest of Asia have quickly recognized the importance of biosafety and biosecurity in swine production over the last 2 yr.

### How to develop a successful plan for ASF prevention and control?

After ASF outbreaks started in China, swine producers quickly learned that, unlike CSF or FMD that could be effectively controlled by mass vaccination, there is no commercial ASF vaccine in the world. Without a tool to implement a “vaccinate-to-live” policy, millions of pigs were culled in the early days of ASF outbreaks in China. Although this control measure seems to be in line with OIE and FAO guidelines, the losses and disruption it created soon became unbearable for at least two reasons: 1) the social and economic impact associated with the huge increase of pork price in a few months after the number of pigs available for the market was reduced quickly and dramatically, and 2) the environmental risk associated with disposing of thousands of pigs on farms in a short period of time.

Without an available safe and efficacious vaccine, swine producers quickly realized that they have to significantly improve biosafety and biosecurity measures on farms to prevent the introduction of ASFV, and use “targeted culling—pull the bad tooth” to remove ASFV infected pigs from the facility to avoid further disease spreading and to preserve the herd. “Reopening” some of the infected farms for production became possible after carrying out intensive disinfection of the infected facility. In addition, significant changes have to be made in biosafety practices to minimize the risks associated with many factors associated with swine production. These risk factors include culled pigs, lagoons, pigs and feed purchased from outside suppliers, selling pigs to others (trucks and personnel from outside vendors), drinking water, boots and coveralls, insects, rodents and pests on farms, swine semen, and use of veterinary pharmaceuticals and vaccines. Implementing policies to incentivize employees to follow biosafety rules and remodeling the current facility for better biosafety control are also common practices for many swine operations. However, many of these changes are very costly and can only be effectively managed by well-funded large operations. Nevertheless, various “Reopening” or “Re-grow” plans have been developed and tested to raise pigs before a highly efficacious vaccine is available.

If a successful plan for ASF prevention and control could be developed, it should resemble the plan that facilitated the U.S. eradication of CSF more than 40 yr ago. Briefly:

1. Know your enemy (the disease and pathogen) through supporting innovative research.
2. Invest strongly and continuously in research related to ASFV, ASFV–host interaction, point-of-contact diagnostics, safe and efficacious vaccines, swine farm biosafety and biosecurity risk management systems, and high containment facilities that are suitable for ASF research.
3. Develop and implement science-based governmental policies at both state and federal levels.
4. Develop and implement science-based animal disease outbreak emergency management policies that will encourage the full participation and support of pork producers and consumers: swine farmers, pork processing plants, and the public. These policies must consider: 1) what will happen if the government does not compensate swine producers for their loss due to ASF outbreaks? 2) how can swine farmers properly cull/dispose of thousands of pigs in a short period of time?, and 3) how do the processing plants/slaughterhouses deal with ASFV positive products?
5. Ensure the cooperation of all stakeholders including pig producers, animal health companies, veterinarians, regulatory agencies, social media, and the public.
6. Because the public is a significant stakeholder of the pork industry, it is not enough to tell the public that ASFV does not infect people. Instead, the swine industry should educate the public that ASF outbreaks affect the livelihood of many parts of the society including swine producers, workers on the farm, grain and feed producers, pork processing plants, grocery stores, truck drivers, animal health companies, restaurants, international and regional pork/grain/feed importers and exporters, and all consumers of pork products. Animal health companies should only manufacture and sell safe and efficacious ASF vaccines, and swine producers should only use authorized ASF vaccines. Veterinarians should employ only field tested, effective immunization, and biocontrol practices. Additionally, ASFV positive products should not be produced, transported, sold, or consumed by anyone. Swine production security is a “weakest-link in the chain” problem. Therefore, the only way to achieve long-lasting security of the system is to improve the strength of the weakest link through full cooperation and regulatory compliance among all stakeholders.

## Future Prospective

CSF and ASF are swine viral diseases with high consequential social and economic impacts in endemic countries. Successful prevention and control of ASF and CSF requires not only safe and efficacious vaccines and fast and accurate diagnostic tools but also science-based government policies that ensure the cooperation of all stakeholders of the swine industry. Science and technology alone are not enough without the effective partnership of the public.

Despite recent devastating outbreaks of ASF and CSF in Asia, the countries of North America and Europe demonstrated decades ago that ASF and CSF can be eradicated with proper government policy and adequate scientific and technological tools. The world has indeed changed since then, notably with ever-increasing high-density swine production and globalization, which demands more innovative approaches to solve new problems:

1. What is the best way to cull/dispose of thousands of pigs in a short period of time in a restricted area to take into consideration of animal welfare, economic and environmental impact, and technical feasibility?
2. Because large quantities of various disinfectants are used to inactivate the ASFV on swine farms, the negative impacts of these biosafety measures on environment, food safety, and human health should be carefully investigated.
3. The transboundary nature of emerging and re-emerging high consequence animal infectious disease threats requires global cooperation not only in outbreak management, but also in research for a broader biomedical, social, and ecological understanding of disease systems.

## About the Authors

**Jishu Shi** is a professor of vaccine immunology at the College of Veterinary Medicine, Kansas State University. He is also a fellow at the Biosecurity Research Institute and the Founding Director of the U.S.-China Center for Animal Health (2010–), Kansas State University. Having worked at Pfizer and multiple academic institutions in the United States in the last three decades, Dr. Shi’s research focuses on novel vaccines and diagnostic assays for high consequence animal diseases including classical swine fever (CSF), African swine fever (ASF), porcine reproductive and respiratory syndrome (PRRS), other emerging viral diseases of swine in the last decade. Several of his research discoveries have been licensed to the animal health industry for product development. In addition to research, Dr. Shi also teaches DVM students and promotes global collaborations in veterinary medicine education through international partnerships among universities, animal health companies, industry associations, and government agencies.

**Lihua Wang** is a research assistant professor at the College of Veterinary Medicine, Kansas State University. He has about 20 yr of research experience in emerging/re-emerging viral diseases, vaccines, and diagnostics. He is the first scientist who discovered that genotype-I Japanese encephalitis virus (JEV) can cause human encephalitis and cocirculation of two genotypes of JEV can lead to epidemic of adult encephalitis. He isolated and identified several novel virus species associated with human or animal disease outbreaks and developed several diagnostic tools for surveillance and control of human and animal viral diseases such as Rabies, classical swine fever (CSF), and African swine fever (ASF). In 2014, he went to West Africa as an infectious disease expert to aid in the fight against the Ebola outbreak in Sierra Leone. He is the coeditor of Modern Plague—Ebola Virus Disease.

**David Scott McVey** is the director of the University of Nebraska Lincoln School of Veterinary Medicine and Biomedical Sciences and the associate dean of the Nebraska/Iowa Program for Veterinary Medicine. Dr. McVey was the Center Director for the USDA ARS Center for Grain and Animal Health Research in Manhattan, Kansas (2017–2020). He was also a supervisory veterinary medical officer and member of the USDA Senior Science and Technology Service. He was previously the research leader for the Arthropod-Borne Animal Disease Research Unit from 2011 through 2016. Prior to 2011, his previous positions were in the biopharmaceutical industry, academia, and private practice. Dr. McVey has been recognized as the 2020 distinguished veterinary immunologist by the American Association of Veterinary Immunologists. He is a diplomat and past president of the American College of Veterinary Microbiologists. Dr. McVey’s research interests include comparative immunology of infectious diseases and associated control measures.
